# Evaluation of the Effect of Different Types of Abrasive Surface Treatment before and after Zirconia Sintering on Its Structural Composition and Bond Strength with Resin Cement

**DOI:** 10.1155/2018/1803425

**Published:** 2018-05-27

**Authors:** Hasan Skienhe, Roland Habchi, Hani Ounsi, Marco Ferrari, Ziad Salameh

**Affiliations:** ^1^Doctorate School for Sciences and Technology, Lebanese University, Beirut, Lebanon; ^2^Research Platform of Nanosciences and Nanotechnologies, Faculty of Sciences, Lebanese University, Beirut, Lebanon; ^3^Department of Endodontics, Faculty of Dental Medicine, Lebanese University, Beirut, Lebanon; ^4^Faculty of Dental Medicine, University of Siena, Italy; ^5^Research Center, Faculty of Dental Medicine, Lebanese University, Beirut, Lebanon

## Abstract

This study evaluated the effect of air abrasion before and after sintering with different particle type, shape, and size on the surface morphology, monoclinic phase transformation, and bond strength between resin cement and zirconia surface using primer containing silane and MDP. Airborne particle abrasion (APA) was performed on zirconia before and after sintering with different particle shape and size (50 *μ*m Al_2_O_3_ and 25 *μ*m silica powder). 120 square shaped presintered zirconia samples (Amann Girrbach) were prepared (3 mm height × 10 mm width × 10 mm length) and polished with grit papers #800, 1000, 1200, 1500, and 2000. Samples were divided into 6 groups according to surface treatment—group A: (control) no surface treatment; group B: APA 50 *μ*m Al_2_O_3_ before sintering (BS); group C: APA 50 *μ*m Al_2_O_3_ after sintering (AS); group D: APA25 *μ*m silica powder (BS); group E: APA25 *μ*m silica powder (AS) at a pressure of 3.5 bar; and group F: APA 25 *μ*m silica powder (AS) at a pressure of 4 bar. Samples were analyzed using XRD, AFM, and SEM. The samples were submitted to shear bond strength (SBS) test. A dual cure resin cement (RelyX Ultimate) and primer (Scotchbond Universal) were used. Data were analyzed with ANOVA and Tukey test (*α* ≥ 0.05). APA in group B significantly increased the surface roughness when compared to all other groups. A significant monoclinic phase transformation (t-m) value was observed in groups C and F and a reverse transformation occurred in presintered groups. The SBS value of group A was 11.58 ± 1.43 and the highest significant shear bond strength value was for groups B (15.86 ± 1.92) and C (17.59 ± 2.21 MPa) with no significant difference between them.* Conclusions*. The use of APA 50 *μ*m Al_2_O_3_ before sintering and the application of primer containing MDP seem to be valuable methods for durable bonding with zirconia. The use of APA 50 *μ*m Al_2_O_3_ after sintering induced the highest (t-m) phase transformation.

## 1. Introduction

Zirconia has been used in dental restoration due to its excellent mechanical properties, biocompatibility, low degree of bacterial adhesion, and acceptable optical properties [1]. The advancement of computer-aided design/computer-aided manufacturing (CAD/CAM) technologies [2] allowed the use of zirconia in a wide range of clinical applications. It is currently used in single crown, fixed partial denture, and implants suprastructures [3, 4]. However, durable bonding of zirconia-based restorations, which is an essential factor for long-term clinical success, has proven to be difficult since hydrofluoric acid etching used with silica-based ceramic has no effect on densely sintered zirconia [5]. This is due to the glass-free composition structure characterizing zirconia as an acid-resistant material. Clinically [6], the most commonly reported complications of zirconia-based ceramics are chipping of veneering porcelains and loss of retention [7, 8]. A durable bond relies on micromechanical interlocking created by surface roughening and chemical adhesion between the cement and ceramic [9]. Adhesive cementation of zirconia restoration is one of the most important factors for achieving clinical success; it improves retention and marginal adaptation and reduces the possibility of recurrent decay [10].

Different roughening methods are applied to promote adequate adhesion between the resin cement and zirconia. The most common method used is sandblasting with aluminum oxide (Al_2_O_3_) particles with different particles shape and size and different abrasive time and pressure [11–13].

It increases the zirconia surface roughness, surface area of bonding, and wettability for micromechanical retention [14]. On the other hand, studies have shown low-bond strength values with air abrasion or even spontaneous debonding after artificial aging [15, 16], and other studies reported decrease in fracture strength of zirconia as a result of surface damage and microcrack formation after air abrasion [17, 18]. It also results in a tetragonal-to-monoclinic (t-m) phase transformation [19, 20].

Other surface treatment modalities that were used as an alternative or in combination with sandblasting, tribochemical silica coating [21], selective infiltration etching [22], experimental hot etching solution [9, 23], laser irradiation [24], Si vapor phase deposition method [25], and nanostructured alumina coating [26] have enhanced the zirconia resin bond strength; however it is not obvious for those methods if it is reliable and could be clinically applicable.

A retentive zirconia restoration relies on the chemical and mechanical bond between resin cement and zirconia surface. Studies [27, 28] have shown that chemical bonding between resin cement and ceramic surface could be achieved by using primer and resin cement based on adhesive monomer containing 10-methacryloyloxy decyl dihydrogen phosphate (MDP) acting as coupling agent; also a recent study [29] reported that the bond between 10-MDP and zirconia was not only ionic bonding, but also hydrogen bonding and high and durable bond strength was achieved on air abraded surfaces with the use of a MDP-containing composite resin [30].

Also recent studies investigated the modification of the zirconia surface in the presintering phase and reported that sandblasting of zirconia before sintering is a useful method to increase surface roughness and, additionally, the monoclinic phase percent of the abraded surface before sintering became zero after sintering [31, 32].

The objective of this study was to evaluate the effect of different zirconia surface treatment on the surface morphology, monoclinic phase transformation, and bond strength between resin cement and zirconia after surface treatment. The null hypothesis was that there was no significant effect of different zirconia surface treatments on the surface morphology, monoclinic phase transformation, and bond strength with resin cement.

## 2. Material and Methods

The materials used in this study are shown in Table 1.

### 2.1. Zirconia Specimen Preparation and Surface Treatment

One hundred twenty rectangle shaped specimens were prepared out of presintered zirconia blocks (Amann Girrbach, Koblach, Austria) using a low-speed diamond disc (Buehler, Lake Buff, Wisconsin, USA); each sample was with 3 mm height, 10 mm width, and 10 mm length. Specimens were polished with silicon carbide grit papers (*Gritflex*, Italy) #800, 1000, 1200, 1500, and 2000. Another 9 specimens were prepared for phase composition analysis of the presintered zirconia. Three specimens were taken from the zirconia block surface as received from the manufacturer without any treatment or cutting (PS), 3 specimens were of cut surfaces (CS), and 3 specimens were taken after polishing with grit paper (GS).

Specimens were divided into 6 groups (*n* = 20) according to the surface treatment performed: (1) Group A: it served as control and specimens did not receive any surface treatment. (2) Group B: the surface of presintered specimens was abraded with 50 *μ*m Al_2_O_3_ particles for 7 s and under 2-bar pressure. The nozzle was placed 3 cm away and perpendicular to the specimen surface. Specimens were then sintered in a furnace (Amman Girrbach) according to the manufacturer's instructions. (3) Group C: specimens were sintered and then air abraded with 50 *μ*m Al_2_O_3_ particles from a distance of 15 mm and at a pressure of 2.8 bar for 10 s. The nozzle was placed perpendicular to the surface of the specimen. (4) Group D: the surface of presintered specimens was abraded with 25 *μ*m high fused silica powder for 7 s and under 2-bar pressure. The nozzle was placed 3 cm away and perpendicular to the surface of the specimen. The blocks were then sintered in a furnace (Amman Girrbach) according to the manufacturer's instructions. (5) Group E: specimens were sintered and then air abraded with 25 *μ*m high fused silica powder from a distance of 15 mm and at a pressure of 3.5 bar for 10 s. The nozzle was placed perpendicular to the surface of the specimen. (6) Group F: specimens were sintered and then air abraded with 25 *μ*m silica powder from a distance of 15 mm and at a pressure of 4 bar for 10 s. The nozzle was placed perpendicular to the surface of the specimen.

After treatment all the specimens were cleaned in an ultrasonic bath of isopropanol for 10 min.

### 2.2. X-Ray Diffraction Analysis

Three specimens from each group were used to evaluate the influence of surface treatment on the crystallographic changes and to determine the phase composition. X-ray diffraction was evaluated using an XRD device (D8 Focus, Bruker ASX GmBH, Karlsruhe, Germany). The surfaces were scanned from 5° to 80° using 2*θ* diffractometer and copper X-unit (Cu-K*α* radiation) 0.02° step scan, at 2 s step interval.

The relative monoclinic to tetragonal peak intensity ratio (*X*m) was based on Gravies and Nicholson's method [33], using the maximum intensities of the reflexes:

(1) $$\begin{array}{c} Xm=\frac{\left\{I_{m}\left(-111\right)+I_{m}\left(111\right)\right\}}{\left\{I_{m}\left(-111\right)+I_{m}\left(111\right)+I_{t}\left(101\right)\right\}} \end{array}$$

*I*_m_  (−111) at 28° 2 theta and *I*_m_  (111) at 31° 2 theta are the intensity of monoclinic peaks and *I*_*t*_  (101) at 30° 2 theta is the intensity of tetragonal peak.

Monoclinic phase volume percentage (*V*m) was calculated using Toraya et al.'s [34] formula:

(2) $$\begin{array}{c} Vm=\frac{1.311Xm}{\left(1+‏0.311Xm\right)}. \end{array}$$

The depth of the transformed layer was calculated using this equation

(3) $$\begin{array}{c} PZT=\left(\;\frac{\sin\theta }{2\mu }\;\right)\left[\;\ln\left(\;\frac{1}{1-FM}\;\right)\;\right] \end{array}$$

where *θ* = 15° (the angle of reflection), *μ* = 0.0642 is the absorption coefficient, and *V*m is volume fraction of m-phase obtained using (1) and (2) [35].

### 2.3. Atomic Force Microscopy

**Figure 1:**
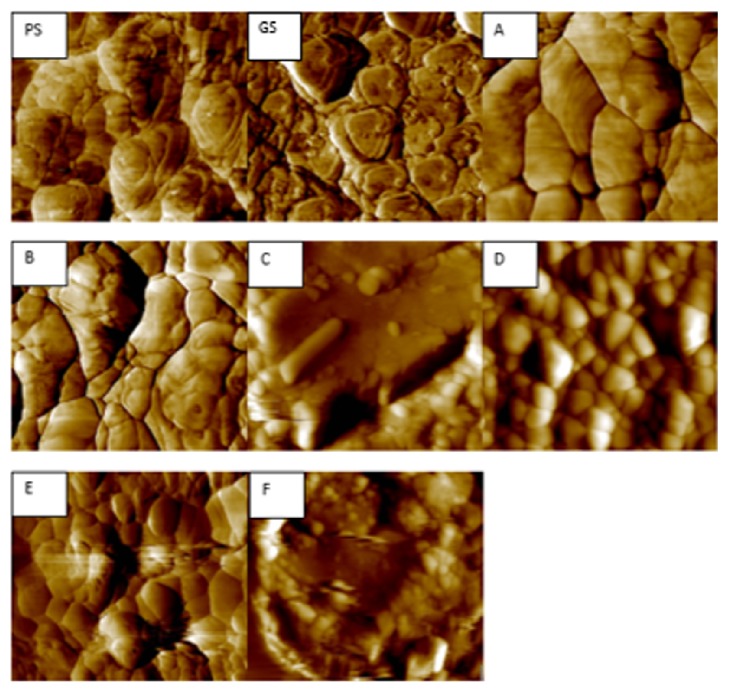
AFM images of groups: PS, GS, A, B, C, D, E, and F. Groups A, B, and D abraded before sintering showed a larger grain with clearly visible grain boundaries. Groups C, E, and F were abraded after sintering; group D only showed clearly visible grains, while groups C and E showed barely visible grain boundaries with smaller grains.

Atomic force microscope (Agilent 5420 SPM/AFM, Agilent Technologies, Santa Clara, CA, USA) analysis was performed in contact mode to detect and observe morphological changes on zirconia surface due to the different surface treatment methods, as well as measuring the surface roughness and grain size. A total of 3 samples for each group were used for surface roughness analysis (Ra), 4 areas on each sample were selected and measured 4 times at different locations, and the mean value was calculated. Twelve AFM images for each group were used for grain size analysis. The mean grain size was measured by Gwyddion software (http://gwyddion.net), by analyzing 72 AFM images (Figure 1).

### 2.4. Scanning Electron Microscopy (SEM) and Surface Elemental Analysis (EDX)

Three specimens from each group were selected randomly, gold sputtered (Sputter Coater 108 Auto, Cressington Scientific Instruments, Watford, UK), and examined using SEM (AIS2100C, Seron Technologies, ASI2100, Gyeonggi-Do, Korea) at 1000x to 3000x magnification and 20 kV. EDX was also performed (AMETEK with EDAX detector).

### 2.5. Shear Bond Strength of Resin Cement

**Figure 2:**
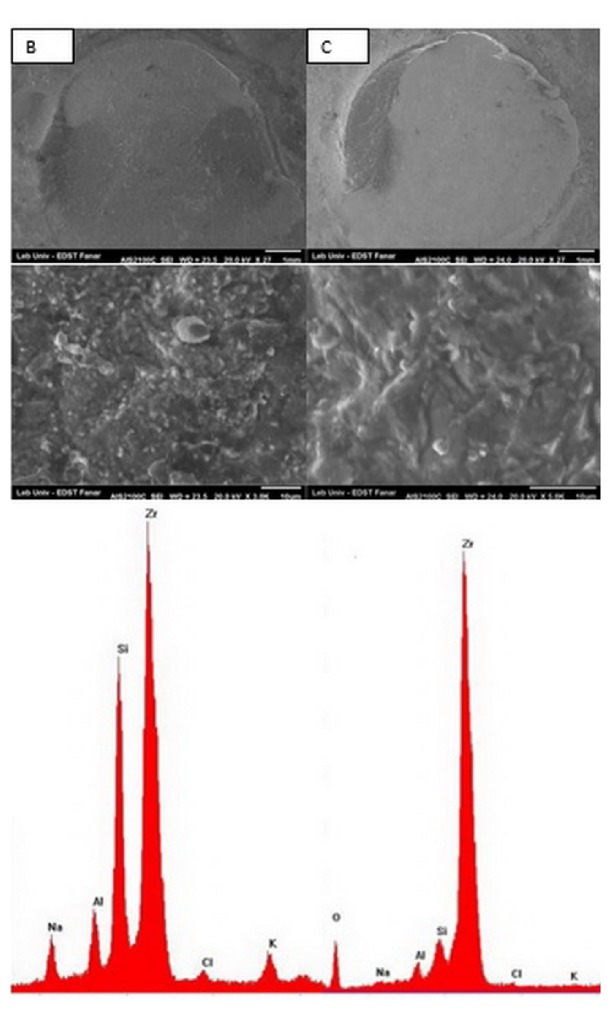
SEM and EDX analysis of groups B and C: failure loads in both groups were mixed and partly cohesive within the composite cylinders.

A Plexiglas mold was fabricated to construct 120 composite resin cylinders (Z 250, 3M ESPE, Saint Paul, MN, USA), 4 mm in diameter and 4 mm in height. The composite was packed inside the mold and light cured using a halogen light (Elipar FreeLight 2 LED, 3M ESPE) for 40 s on the top and then 40 s on the bottom surface of the resin cylinders. A ceramic primer (Scotchbond Universal, 3M ESPE) was applied on the surface of the zirconia sample using a microbrush, left for 20 s, then dispersed using dry air for 5 s, and left to react on the zirconia surface for 180 s. Dual-cured resin cement (RelyX Ultimate, 3M ESPE) was mixed and applied directly onto zirconia surface following the manufacturer's instructions. The composite cylinders were seated on the zirconia surface and a fixed 500 g load was applied for 50 s perpendicular to the surface of the composite cylinder using a custom made device and excess material was immediately removed using a microbrush. Light polymerization followed laterally at the interface area for 40 s from 3 different directions. Then the bonded samples were stored in distilled water at 37°C for 24 h. The bonded samples were mounted on a universal testing machine (YL-UTM Main, YLE GmBH, Bad König, Germany). A chisel semicircle indenter was used to direct the shearing force as close as possible to the zirconia composite interface at a crosshead speed of 1 mm/min until failure occurred. The load was recorded in Newtons and converted to MPa by dividing it by the surface area (*a* = *P*/*A*). All failed samples were analyzed using SEM to evaluate the fracture pattern, if it is failed cohesively between resin and zirconia or adhesively within the resin cement or mixed. Then EDX analysis was done for failed samples (Figure 2).

### 2.6. Statistical Analysis

The data were collected and grouped for statistical analysis using a statistical software package (SPSS version 23, CITY, USA). One-way analysis of variance (ANOVA) was conducted to evaluate the null hypothesis, followed by post hoc tests for multiple comparisons Tukey test, *α* ≥ 0.05. Statistical significance was set at 0.05.

## 3. Results

### 3.1. X-Ray Diffraction Analysis

Three samples from each group were used for X-ray Diffraction Analysis. The average *X*m% of the treated groups was compared with the average of control. Surface treatment applied on zirconia surface and its effect on phase transformation with significant difference (*p* < 0.05) were shown in Table 2.

The mean *X*m% of the PS was 6%, and for CS it was 13%. After polishing by grit paper, the mean *X*m% of GS samples became 6% again. The mean *X*m% of group B that was abraded before sintering was 14%; after sintering *X*m% became zero.

The volume fraction of monoclinic phase of the studied groups is shown in Table 3.

The transformed depth zone (TDZ) appeared to be directly related to the *X*m%; groups C and F had higher TZD than all other treated groups (Table 3).

**Figure 3:**
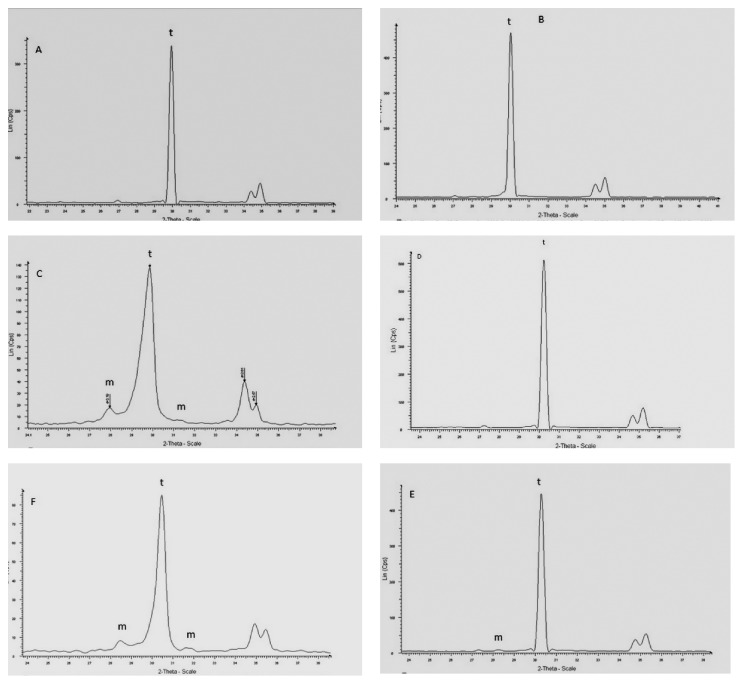
XRD of the studied groups A to F: groups C and F showed higher *X*m%, reversed intensity of the tetragonal peaks t (002) and t (200), and a hump on the left shoulder of (101) t peaks. Groups C and F show decrease of the intensities of tetragonal peak (101).

An asymmetrical broadening and decrease of the intensity of tetragonal peak t (101) in groups C and F had the highest *X*m% (Figure 3). In addition, an increase of the FWHM (full width at half maximum height) and reversed intensity of the tetragonal doublets were corresponding to (002) and (200) planes (Table 3).

### 3.2. Surface Roughness and Grain Size Evaluation

The value of Ra in *μ*m was chosen as the indication of surface roughness. The lower means Ra value was for control group A. The highest Ra value was for group B followed by groups C, F, D, and E. The mean values of grain size and surface Ra with significant difference are shown in Table 2. The mean grain size of the polished samples before treatment and sintering (GS) was 0.12 *μ*m; after sintering it became 0.32 *μ*m. Groups C and F showed a significant decrease (*p* < 0.05) of mean grain size (0.20 *μ*m and 0.19 *μ*m), respectively, when compared to control group (0.32 *μ*m) as well as to groups B and D (0.31 *μ*m and 0.30 *μ*m). The AFM image showed that groups A, B, D, and E had a clear grain distribution and borders, while groups C and F showed a barely visible grain shape and boundaries.

### 3.3. SEM and EDX

**Figure 4:**
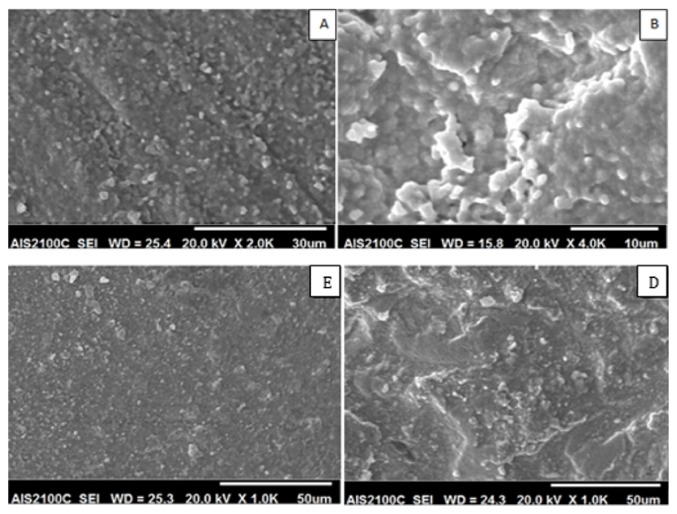
SEM of groups A, B, D, and E. Groups A, B, and D treated before sintering appeared with deep grooves, round elevations, and grain coarsening. Abrasion of group E by silica particle after sintering did not change the surface texture.

**Figure 5:**
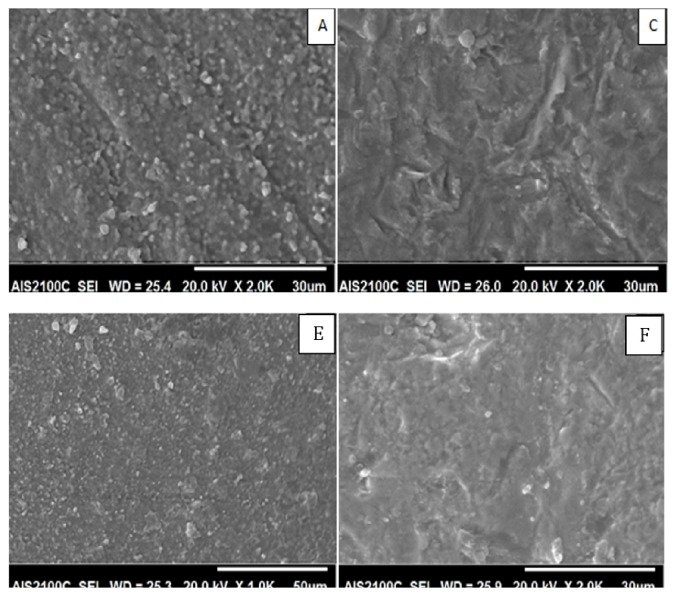
SEM of groups A, C, E, and F. Group C abraded after sintering by 50 *μ*m Al_2_O_3_ particles and group E abraded after sintering by 25 *μ*m silica particles with 4-bar pressure appeared with pits, grooves, and sharp edges, while group D abraded by 25 *μ*m silica particles after sintering with 3.5 bar appeared as control group.

The SEM images of the treated zirconia specimens are presented in Figures 4 and 5. According to SEM images, the surface morphology of all groups was different from that of control group except group E. SEM image of group B was different from other groups; it appeared like a 3D image with deep grooves, round elevations, and small particles projection. In groups C and F scratches with sharp edges and pits were formed, but they appeared extensively in group C. Group E images showed small particles projection, but they are devoid any grooves. EDX analysis showed no impacted alumina or silica particles in any group.

### 3.4. Shear Bond Strength of Resin Cement

Table 2 shows the means and standard deviation (SD) of shear bond strength values with significance difference. The highest value was for group C (17.59 MPa) followed by group B (15.86 MPa) with significant difference with all other groups, while no significant difference exists between groups B and C. The failure mode in groups A, D, E, and F was adhesive failure, while for groups B and C it was mixed. Figure 2 shows the SEM image and EDX of the failed surface of groups B and C.

## 4. Discussion

The result of the present study led to the rejection of the null hypothesis that the phase transformation of zirconia ceramics will be changed according to the surface treatment applied, since the type and size of abraded particles and the pressure that was used increased significantly the monoclinic percent.

The extent of morphological changes on zirconia surface and t-m phase transformation depends on the particles size and blasting pressure [11, 20, 36]. In the present study, the size and type of abrasive particles could be the dominant factors for t-m phase transformation. This appeared through the statistical comparison between group C that was abraded by 50 *μ*m Al_2_O_3_ particles which had the highest significant t→m phase transformation 11% and group E that was abraded by 25 *μ*m silica particles with higher pressure than group C which had the lowest significant t→m phase transformation. The impact of harder and larger alumina particles on zirconia surface could explain the reason for highest *Xm* in group C; this could be confirmed by AFM images observation (Figure 1), showing clear grain boundaries in group E, while barely visible grain boundaries in group C; also the grain size value of group C was significantly lower than that of group E.

Hallmann et al. stated that the grain boundaries were visible with 50 *μ*m alumina abrasion and 2 bar, while they disappeared with 110 *μ*m and 1.5-bar blasting pressure [20].

Controversially, in this study the AFM image showed that the grain boundaries were not visible only in groups C and F.

In the present study, the lower-energy abrasive silica particles with reduced size were used to reduce the damage that could occur from hard and coarse abrasive particle, and the pressure selection in groups E and F was based on pilot evaluation that started with the same pressure used in group C (2.8 bar), but at this pressure the SEM and XRD analysis showed no effect regarding the morphological changes and phase t-m phase transformation. Consequently the pressure that was used is the lower one that begins to induce the t-m phase transformation followed by the highest one that induced t-m phase transformation. So this selection was to investigate the effect of pressure on phase transformation and the suitability of silica particle to be an alternative abrasive material. By comparing the results of group C and groups E and F, it was shown that the hardness was the principal factor for t-m phase transformation, since the 3.5 bar in group E and 4 bar in group F induced t-m phase transformation smaller than that induced by 50 *μ*m alumina with 2.8-bar pressure (Figure 3).

However the comparison between groups E and F that were abraded with 25 *μ*m silica particles with the same time and distance but with different pressure revealed that pressure was the principal factor for t-m phase transformation, since 4-bar blasting pressure in group F was needed to induce 8% t-m phase transformation while 3.5 bar for group E induced 2% t-m phase transformation.

Moon et al. stated that the t-m phase transformation increased with larger particle size and higher pressure [11]. On the contrary, Chintapalli et al. stated that 12 and 15% monoclinic phases were found after air abrasion irrespective of the particle size and pressure, and the changes in particle size and pressure have small effect on the phase transformation due to erosion of material [37], although in their study they did not mention the time of abrasion and its effect on erosion.

TZD values correspond to the protective layer against residual compressive stresses that is created as a result of t→m phase transformation on the Y-TZP surface; it is directly linked with an increase in the mechanical resistance of zirconia [21, 26]. Amaral et al. stated that the defects that result from air abrasion appeared to remain confined within the transformation layer, where they were probably healed by the 4% volume increase in the grains during the phase transformation [38]. Kosmac et al. found 15–17% t-m phase transformation that yielded the TZD values ranging from 0.3 *μ*m to 0.5 *μ*m [39].

The results of the present study showed that the calculated TZD was directly related to the percentage of monoclinic phase transformation (Table 3). The maximum TZD of group C that had 11% t-m phase transformation was 0.21 *μ*m, and group F that had 8% t-m phase transformation was 0.10 *μ*m. These results revealed that the t-m phase transformation was restricted to the surface grain layer, as the mean grain size of sintered polished samples before air abrasion was found to be 0.32 *μ*m.

Thus, the compressive stress generated by transformation toughening on the material surface appeared to counteract any possible deleterious effects associated with sandblasting [9, 38], whereas with the progression of monoclinic phase transformation from the surface to the bulk of the material microcracks and tensile residual stresses may develop and decrease the flexural strength [38].

The present results were in agreement with studies that stated that the increase of phase transformation will lead to deeper TZD to the bulk of zirconia [38–40].

The diffraction pattern of groups C and F showed an asymmetrical broadening and decreased intensities of the t (101) peak and increase in full width at high maximum height (FWHM), as well as a reversed intensity of the tetragonal peaks t (002) and t (200) and a hump on the left shoulder of (101) t peak. These observations were related to residual compressive stresses due to the type and size of abrasive particles in group C, as well as the type and pressure that were used in group E. Other factors that contribute to this broadening are the cubic phase at c (111) in the place of the most intense peak t (101) and the grains broken under stress that may exist in the upper surface layer [41].

This was confirmed by the comparison that was done between group F (4 bar) and group E (3.5 bar), which revealed that the diffraction pattern of group E showed no changes.

Hallmann et al. stated that the reversibility was dependent on the abrasive particle size and blasting pressure, and the increase in FWHM depended on several factors, one of them being lattice strain [20].

In the present study, a reverse m-t phase transformation occurred in groups A, B, and D as a result of sintering process, and the *X*m% present in presintered samples was almost zero after sintering. This agrees with many studies stating that monoclinic phase present in presintered samples was integrally transformed into tetragonal phase [31, 32, 42].

Regarding the bond strength, air abrasion, and primer, the null hypothesis was also rejected as the control group had the lowest significant RA value. This was also the case for the use of air abrasion and primer as they significantly increased the bond strength with resin cement.

The SBS value of group C was higher than that of group B, but with no significant difference despite having highest RA value in group B, whereas group E had the lowest shears bond strength value despite having the same RA value as group C. Therefore, those results revealed that the surface roughness was not the responsible factor for higher bond strength in group C.

Several studies stated that micromechanical retention was not affected by the abrasive grain size (25 *μ*m, 50 *μ*m, or 110 *μ*m) used despite the difference in surface roughness created [43–46], while others observed improvement of bond strength with resin cement with smoother surface produced after air abrasion with 50 *μ*m alumina particles [47, 48].

Therefore one of the factors that could be responsible for the highest RA value of group B could be related to the effect of hard alumina particles on soft presintered zirconia surface that was greater than its effect on densely sintered surface. These results were in agreement with previous studies that stated that abrasion of presintered samples had the highest mean RA value [31, 32, 42], whereas other reports stated that the shrinkage associated with grain growth and monoclinic to tetragonal phase transformation could have contributed to the increased roughness [49–53].

The lower shear bond strength value of group D that was abraded by 25 *μ*m silica particles could be due to the loss of the microretentive grooves during sintering as a result of increase in grain size and sintering shrinkage; thus it lost a bigger part of its surface microretentive pits and behaves as a smooth surface and consequently lost a part of its surface area of bonding.

But when compared to group B which is also subjected to the same phenomenon and had the highest RA value and a higher significant SBS value, it preserved a part of its bigger grooves and pits, since the grooves created as a result of abrasion by 50 *μ*m alumina particles were bigger than that created in group D. Accordingly, group B had a greater surface area of bonding resulting from increased grain size and deep valleys sustained after sintering; this could be one of the reasons for increased SBS value.

This explanation was in agreement with a recent study by Retamal et al. that stated that zirconia superplastic properties can be explained by grain boundary sliding of plane interface and grains gliding against each other keeping a compact structure [54]. Other studies confirmed as well that the average grain size of 3YTZP ceramics increases with sintering temperature [55, 56]. This was also observed by AFM images (Figure 1) that showed a clear grain distribution only in the control group and in groups that were abraded before sintering. The clear grain boundaries were due to increase of grain size as a result of the sintering process.

The irregularities that were created in group C increased the surface area of bonding and improved the wettability, allowing the resin or primer to flow into this surface. This could explain the highest SBS value in group C. Our results were in agreement with studies that stated that the dominant mechanism to obtain durable, long-term bonding to dental ceramic and resin agent was micromechanical retention [27, 57].

The use of Scotchbond Universal primer and RelyX Ultimate in combination with air abrasion using alumina particles created a significant increase in SBS in groups B and C. Therefore air abrasion and the chemical reaction between primer and zirconia surface were the dominant factors that increased the SBS value. Those results were in agreement with a recent study for Nagaoka et al. that evaluated the chemical interaction and the bond strength between MDP and zirconia; they found that the bond between 10-MDP and zirconia was not only ionic bonding, but also hydrogen bonding; as well they stated that the 10-MDP monomer could be adsorbed onto the zirconia particles via hydrogen bonding or ionic interaction between the P-OH and Zr-OH groups or between P-O− and partially positive Zr, respectively [29].

The SBS data of groups that was abraded by silica particles showed no significant increase in SBS value despite having the same RA value as group C. Therefore those results revealed that the abrasion of hard zirconia with silica could not lead to a viable surface for micromechanical interlocking.

The important factor in estimating the long-term performance of the material is aging, since mechanical stresses, temperature, and humid environments can influence the degradation of the strength zirconia [13, 14] and the durability of bond with resin cement. Chen et al. concluded that increase in the m–ZrO2 phase is unlikely to contribute to reduction in bond durability. Rather, it is the hydrolysis of the coordinate bond between MDP and zirconia that is responsible for the deterioration of the integrity of the bond between MDP-conditioned Y-TZP and methacrylate resin [58].

Aurelio et al. stated that there are contradictory findings in literature regarding the increase and decrease of the mechanical strength of zirconia as a result of phase transformation created due to air abrasion, which may result from the different protocols used, with variations in particle size and pressure, as well as the presence or lack of aging conditions [59].

Previous studies reported that that crack growth by the degradation mechanism of zirconia in water has a direct association with the failure of restorations [1, 60] and the cyclic fatigue in water was shown to present a high impact on the lifespan of different zirconia materials yielding to significantly lower results than when mechanical cycling was performed in dry conditions [61].

In contrary, Amaral et al. stated that the increase of flexural strength obtained for the air- abraded group is probably related to the tetragonal-to-monoclinic phase transformation [12]; as well studies stated that the formation of protective compression layer on zirconia surface may decrease the detrimental effects of aging on the specimens [39, 62, 63] as the ~4% expansion in the volume of grains obstructs crack propagation [38, 39] and thus prevents the drop in the flexural strength.

However, although the TZD in the present study exists within the surface grain layer and does not extend to the bulk of zirconia, abrasion of densely sintered hard zirconia ceramic for roughening purpose could be detrimental and could compromise the mechanical properties of zirconia, through the progress of the transformation which could lead to grain pullout and surface degradation from the applied oral stresses, leading eventually to the failure of restoration [17, 64].

Regarding the surface treatment of group B, the reverse m-t phase transformation [42], sintering shrinkage, and the increase in grain size [55, 56] could aid in partial or total sealing of the cracks that could be resulting from presintered surface treatment.

Abi-Rached et al. stated that flexural strength of group abraded before sintering behaved like the nonabraded group [32], while Denry stated that residual stresses of mechanical or thermal origin and subcritical crack growth play a key role in the mechanical performance of partially stabilized zirconia [52].

Based on the results presented, it can be stated that air abrasion with 50 *μ*m alumina particles before sintering could be a simple and valuable surface treatment method when compared to air abrasion with 50 *μ*m after sintering, since it can introduce a zirconia surface free of t-m phase transformation and compressive stresses. It remains however that the effect of presintered surface treatment on mechanical properties of zirconia needs more investigation as well as the effect of aging on the bond strength and flexural strength for both pre- and postsintering abrasion, which could be considered a limitation in this study. As well, regarding the surface roughness, the topography in *z* direction could far exceed the cantilever tip range of AFM; it could be considered another limitation in this study.

## 5. Conclusions

Within the limitations of this in vitro study the following conclusions can be drawn:

1. The combination of micromechanical and chemical surface treatment is prerequisite for a durable bond with zirconia ceramics.
2. The abrasion of hard zirconia with silica could not lead to a viable surface for micromechanical interlocking, while air abrasion with 50-micron alumina particles after sintering increased significantly the SBS, but with highest t-m phase transformation.
3. Air abrasion with 50-micron alumina particles before sintering could be a simple and valuable surface treatment method, since we can introduce a restoration with zero monoclinic phase and minimal compressive stress.
4. The size and type of abrasive particles as well as the pressure were major factors for micromechanical retention.

## Figures and Tables

**Table 1:** Materials used in this study.

Material	Composition	Lot/Manufacturer
Zirconia/*Ceramill Zi*	Yttria stabilized polycrystalline tetragonal zirconia (Y-TZP) blank (ZrO2 + HfO2 + Y2O3 99.0)	1512006-1/Amann Girrbach/Koblach/Austria

Primer/Scotchbond Universal	MDP/Vitrebond copolymer/HEMA/silane/ dimethacrylate resins/fillers/initiators/ethanol	635860/3M ESPE, Germany

Resin cement/RelyX Ultimate	Base: Methacrylate monomers/radiopaque, silanatedfillers/initiators/stabilizers/ Rheological additives Catalyst: Methacrylate monomers/ radiopaque, alkaline fillers/initiators/stabilizers/pigments/ Rheological additives/fluorescence dye/dark cure activator for Scotchbond Universal adhesive	636403/3M ESPE, Germany

Composite resin Filtek Z250, shade A1.	(Bis-GMA, UDMA, Bis-EMA, ZrO2/SiO2)	762333/3M ESPE, USA

**Table 2:** Mean values of groups for surface roughness (RA), grain size (GS), shear bond strength (SBS), and monoclinic percent (*X*m). Similar superscripts indicate no significant difference.

GROUPS	RA *μ*m	GS *μ*m	SBS MPa	*X*m%
A	0.05 ± 0.02^b,c,e^	0.32 ± 0.07^c,f^	11.58 ± 1.43^b,c,f^	0
B	0.1 1 ± 0.06^a,c,d,e,f^	0.31 ± 0.09^c,f^	15.86 ± 1.92^a,d,e,f^	0
C	0.08 ± 0.04^a,b^	0.20 ± 0.04^a ,b,e,d^	17.59 ± 2.21^a,d,e,f^	11^a,b,d,f^
D	0.07 ± 0.03^b^	0.35 ± 0.06^c,f^	12.07 ± 1.11^b,c,e,f^	0
E	0.07 ± 0.02^b^	0.30 ± 0.06^c,f^	10.21 ± 1.17^b,c^	2^c,e^
F	0.08 ± 0.03^a,b^	0.19 ± 0.03^a,b,e,d^	9.46 ± 2.75^a,b,c,d^	8^a,b,d,e^

**Table 3:** Mean values for FWHM, *X*m%, *V*m, and TZD. Similar superscripts indicate no significant difference.

Groups	AV-FWHM t (111)	Av *X*m%	*V*m	TZD *μ*m
GS.BS	0.45	6	2.74	0.12
D. BS	0.27	5	2.56	0.10
B.BS	0.25	14	3.42	0.30
A	0.26	0	0	0
B	0.29	0	0	0
C	0.62	11^a,b,d,f^	3.26	0.23
D	0.28	0	0	0
E	0.30	2^c,e^	1.61	0.04
F	0.41	8^a,b,d,f^	3	3.24

## Data Availability

The data used to support the findings of this study are available from the corresponding author upon request.
